# The effect of electro-magnetic-energy-regulation therapy on subjective sleep among elite players in Norwegian women's football

**DOI:** 10.3389/fspor.2024.1343841

**Published:** 2024-08-01

**Authors:** Frode Moen, Svein Arne Pettersen, Ellen F. Mosleth

**Affiliations:** ^1^ Department of Education and Lifelong Learning, Faculty of Social and Educational Sciences, Norwegian University of Science and Technology, Trondheim, Norway; ^2^ The Norwegian Olympic Sport Center, Trondheim, Norway; ^3^ School of Sport Sciences, Faculty of Health Sciences, UiT, The Arctic University of Norway, Tromsø, Norway; ^4^ Nofima AS, Ås, Norway

**Keywords:** sleep, elite football, recovery, physical load, BEMER therapy

## Abstract

The current study investigated if Bio-Electro-Magnetic-Energy-Regulation (BEMER) therapy is affecting subjective sleep among a sample of 21 elite female football players in a Norwegian top series club. Subjective sleep was measured each day over a period of 273 consecutive days by using a smartphone application, PM Reporter Pro, which scheduled push messages to remind the participants to report sleep every morning. The study was conducted as a quasi-experimental design, with a control period before the introduction of BEMER therapy that lasted for 3 months, followed by an intervention period where BEMER therapy was used that lasted for 5 months. The collected data from the players in the control period served as their control compared to the data collected from the players in the intervention period. Analyses of variance (ANOVA) with False Discovery Rate adjusted *p*-values show that subjective sleep duration and subjective sleep quality are significantly reduced on game nights, both in the control period and in the experiment period. The results also show that subjective sleep duration and subjective sleep quality significantly increase in the experiment period compared to the control period. The findings indicate that BEMER therapy might serve as a tool to improve sleep.

## 1 Introduction

Sleep is the most crucial state of the body to gain optimal restoration and recovery of virtually all major systems of the body that are required for athlete progression in sport (1–3). Both physical, neural, and psychological restoration happen during effective sleep (4–6), and sleep has consequently been recognized as a crucial part of recovery for elite athletes in sports (7–9). An athlete's perceived effectiveness and quality of own sleep and daytime functioning are defined as the person's sleep quality (10). Thus, sleep quality is high when the athletes gain both the needed duration of sleep and an appropriate distribution of the different sleep stages during the night (11). Importantly, the need for sleep is associated to the daytime loads the athlete is exposed to, and the major systems of an athlete's body are restored when sleep quality is high (12).

Athletes in elite sports are often exposed to both physical and mental loads that are close to or on the absolute limits of their tolerance (2, 13, 14). Therefore, elite athletes' sleep is found to be crucial (15). Sleep is especially found to be important in elite football (soccer) because of the extraordinary cognitive, emotional and physical loads that are associated with elite football games (16, 17).

Interestingly, the physical demands in women's elite football have significantly increased the last decade and the physical actions need to be both more powerful and faster (18–22). Faster movements causes players to perceive the movements of team and opponent players more rapidly, make faster decisions in the play-counter play, and execute the needed actions to outplay the opponent team faster (23, 24). Thus, the cognitive loads in women's elite football especially peak on game days caused by the faster shifting movements among team- and opponent players (17, 25). The physical loads are also found to significantly peak on game days because of the high occurrences of demanding actions such as sprinting, sudden changes in movement directions, accelerations and decelerations, powerful tackles, and maximal jumps (26–29). Such considerable physical loads often lead to muscle fiber micro ruptures and muscular fatigue (30), and the cognitive loads might lead to perceptual-cognitive overload (19). Such a peak in physical and cognitive loads leads to increased need for recovery (31, 32), and such loads, especially associated to women's elite football games, require substantial recovery time (33, 34).

However, physical and cognitive efforts associated to football games might lead to disturbances in the players' physiology and neural processes, and might negatively affect sleep (17, 29). Particularly football games are found to negatively affect the following nights of sleep (27). Studies show that football games reduce both the players' sleep duration and their sleep quality (17, 34, 35). One study shows a reduction of 65 min in sleep duration after women's elite football games, when the researchers compared the players' baseline sleep on ordinary training days with their sleep on game nights (36). Thus, the paradox is that the the need for recovery increases significantly after games in elite women's football, but sleep is found to be disturbed following game days (27, 33, 37–39).

Interestingly, extended sleep is found to have positive effects on athletes' performances (40, 41), and poor sleep impairs several aspects of physical and cognitive athletic performance, including increased risk of injuries (42). Therefore, methods and tools that can help the players to gain more effective sleep are therefore desirable in elite women's football (1, 17, 43).

A recent study found that Electro-Magnetic-Energy-Regulation therapy (BEMER) positively affected women's objectively measured sleep on game nights and nights associated to game nights (27). The players' total sleep time, time in bed, light sleep, and REM-sleep significantly increased after the introduction of BEMER therapy, and the players who spent more time with BEMER therapy compared to the players who spent less time, significantly increased their total sleep time, sleep efficiency, light sleep, light sleep, REM-sleep, and decreased their sleep onset latency and wakenings after sleep onset (27). The findings are explained by the possible restorative function BEMER has on recovery because of increased microcirculation.

BEMER therapy uses a Pulsed Electromagnetic Field (PEMF) to influence human recovery through a physical vascular therapy (44–47). The PEMF therapy is found to affect the blood flow in capillaries (arterioles and venules) which is defined as vasomotion (48, 49). The microcirculation of the body is the transportation of blood in the smallest blood vessels such as capillaries, arterioles and venules, which enable the blood flow through a contraction-extraction system within the tissue in the blood vessel walls (27). BEMER therapy sends a low intensity pulsed electromagnetic field into the body in order to stimulate the contraction-extraction frequency that regulates the local blood flow. The microcirculation system is crucial in the restoration and recovery of the cells in the body (50, 51). It is the microcirculation that enables the blood cells to deliver both oxygen and nutrition to organs and cells in the body, remove waste materials (such as carbon dioxide) from organs and metabolism, a process needed to rebuild the cells after extraordinary loads (52, 53). Interestingly, when the body is exposed to substantial stress loads, vasomotion is found to be hampered (54). Thus, tools that can affect vasomotion effectively might be beneficial for recovery in athletes (55–57). While BEMER therapy was found to affect objectively measured sleep on game nights and nights related to football games among female elite players, there is no such findings from BEMER therapy on subjective reported sleep to the authors knowledge (27). Despite the known importance of sleep for athletic performance, there is limited research on effective interventions to enhance sleep duration and sleep quality among elite women's football players. The current study therefore aims to investigate if women's elite football players subjective measured sleep duration and sleep quality are affected by BEMER therapy.

The following hypothesis were developed:

*H^1^ BEMER therapy is affecting the elite women's football player’s sleep duration and sleep quality on game nights and the nights following game days.*

## 2 Method

### 2.1 Participants

The current study is one of several studies based on a data collection in a Norwegian top series team in women's football (27). The team was selected and invited to participate in the current study based on their cooperation with the Olympic Sport center in middle Norway. The Olympic Sport center works with elite coaches, elite athletes and elite teams, to help them become international elite athletes. The coaching staff were first informed about the research project, where the aim of the study was explained and discussed in detail. The coaching staff agreed to participate based on their belief that the intervention might affect the recovery of the players. The team of 25 players were then informed about the project and the players who agreed to participate were instructed to sign a consent form approved by the local Regional Committee for Medical and Health Research Ethics (REC) in Central Norway (project ID 2017/2072/REK midt). Out of the team of 25 players, twenty-one football players (mean age 23.68 ± 2.8, range 20–29 years) signed the consent form and agreed to participate in the current study.

### 2.2 Procedure

Once the players were enrolled in the study the players were instructed about the data collection process and timeline. For the current study data were collected by day-to-day reports from the players of their subjective sleep. The data collection of day-to-day reports was reported by a smart phone application, PM Reporter Pro sport logging system (58), and the collection started on the 1st of February and lasted until the 31st of October the same year. The current study was designed as a quasi-experiment, where each player was their own control based on a control period compared with an intervention period. The first period of the study, from the 1st of February until the 30th of April, was the control period (Exp1) where the players completed their normal routines with trainings, games, and normsl recovery, but no use of BEMER therapy. The period from 1st of May until 31st of May was the period where the players were introduced for the intervention and got familiarized with the BEMER therapy device (Exp2). The intervention included BEMER therapy each morning and evening, and after high intensity trainings and games. The period from 1st of June until the 31st of October was the experiment period. However, because of extraordinary loads due to qualifying games for the Champions League and play offs in the Norwegian national cup in October, this period was divided into two periods; from 1st of June until the 30th of September (Exp3) where players completed training and games that were comparable with the control period (Exp1), and from the 1st until the 31st of October (Exp4) where players were exposed to extraordinary loads that were not comparable with the control period.

### 2.3 Instruments

The day-to-day data collection of subjective sleep was collected by using a smartphone application, PM Reporter Pro, which is a part of the PMSys online sports logging system (58). The application scheduled for push messages to remind the participants to report data every morning on a daily basis.

#### 2.3.1 Subjective sleep

Subjective sleep duration and sleep quality were reported by the players in the morning and the following variables were obtained from the online sport logging system; Subjective sleep duration (SSH, “How much did you sleep last night?” hours and ½ hours), and Sleep quality (SLQ, “How well did you sleep?” 1 = insomnia, 5 = excellent sleep).

#### 2.3.2 Subjective effects

The players also completed a questionnaire that registered their subjective experience of possible effects from the BEMER therapy. The soccer players were asked to consider 3 questions and how satisfied they were with their experienced effect on their restitution (Subjective restitution-SRC), their sleep (Subjective sleep-SSL), and their experienced effect on their experienced muscle sourness (Subjective muscle sourness-SMC), on a 7-point scale ranging from 1 (not at all satisfied) to 7 (extremely satisfied).

#### 2.3.3 BEMER therapy

Bio-Electro-Magnetic-Energy-Regulation (BEMER) uses a pulsed electromagnetic field (PEMF) to deliver a patented bio-rhythmically therapeutic signal during the therapy. Each of the players in the current study were equipped with a BEMER Essential-Set that they had at home at their residence. The set contains a BEMER Box Professional control unit, where the football players administrate the different therapy programs that were used during the intervention, the BEMER Body applicator for full-body treatment, and the BEMER Pad applicator for local body treatment. The BEMER Box Professional control unit calculates the usages in minutes and hours each therapy program was used by the players to detect the total amount of minutes and hours each soccer player used BEMER therapy during the intervention period. The BEMER therapy was conducted by using the BEMER Body applicator (a mattress for a full body treatment) and a BEMER Pad applicator (a belt for local treatment).

#### 2.3.4 The intervention protocol

From the 1st of May the players were instructed to use the BEMER base program on a day-to-day basis for 8 min in the morning and 8 min in the evening (27). All players had their own BEMER set. The intensity was set to 1 the first week and increased with one intensity for each week for the first 6 weeks. Then the intensity was set back to 3 and increased with one each week until they reached intensity 6, and then back to intensity 3 again. This routine was followed throughout the rest of the intervention period. The players were also instructed to use the BEMER therapy program after high intensity training session and games. Level 2 was used for the first two weeks followed by level 3 the next weeks throughout the intervention period. The BEMER Special mode program was introduced to the players after finishing 8 weeks with BEMER therapy treatment. The BEMER Special mode program starts treatment 2 h before players' awakenings, and was used once the first week, twice the second and up to three times a week throughout the intervention period.

### 2.4 Statistical analyses

Demographics and descriptive statistical analyses were conducted initially with IBM SPSS (version 27.0) and results were presented as means and ± standard deviations (S.D.). The days/nights in the data collection period were classified as training day/night (TN) if the players completed an ordinary training session that day, and that the day/night was not close to a football game. Days/nights that were close to a football game were classified as night before game (NBG) if the night was the night before a football game, game night (GN) if the night was the night following the day of the football game, game night plus 1 (GN1) if the night was the night one night following game night, and game night plus 2 (GN2) if the night was two nights following the game night. Each date of the night associates to the date of the day where the need for recovery and sleep are developed. Thus, the date of training or game day equals the date of sleep night, regardless of if sleep starts before or after midnight. Dates of days/nights without observations were omitted for further analyses.

The players' means of general training days/nights (TN) were calculated as their baseline values in each period of the experiment (Exp1, Exp2, Exp3 and Exp4), and their values on nights close to football games (NBG, GN, GN1 and GN2) were used to calculate possible differences to their baseline values in each period of the experiment. The period in the experiment where the players experienced extraordinary loads that were not comparable with the control period (Exp4), there were occasions of successive games with only one day between and significant data loss. Data from this period was therefore omitted from further analyses.

The different classified types of nights were classified within each of the periods in the experiment (Exp1, Exp2, Exp3 and Exp4). Accordingly, the players in the experiment completed BEMER therapy treatment in various degree, and the real time each player completed therapy treatment was collected from their BEMER Box Professional control units at the end of the experiment (after 31st of October). The actual time the players were exposed to BEMER therapy treatment was defined as BEMER Time (BMT). For further analyses the types of nights were categorized as the variable Type, the different periods in the experiment, control period (Exp1) and experiment periods (Exp2, Exp3 and Exp4) were categorized as the variable Exp.

The subjective sleep data (SSH and SLQ) and the category variable Exp was analyzed as three-ways factorial design by univariate analysis of variance ANOVA with False Discovery Rate adjusted *p*-values (59) and by multivariate analysis of variance using FFMANOVA (60, 61). The two category variables and the two-ways interactions between them were used as design variables in the analyses. The analysis was performed in R version 4.2.1 using the FFMANOVA R package (https://cran.r-project.org/package=ffmanova) (62).

## 3 Results

The football players participated in the experiment over 273 days, resulting in a total potential of collecting 6,006 data points including subjective sleep duration and subjective sleep quality. Of the 6,006 potential data points of perceived sleep data, 4,440 or 73.9% were collected. Data was lost due to the players' occasional forgetfulness, and due to the daily demands to report the soccer players' subjective sleep over such a long period, and that one of the soccer players was sold to another club in July.

Descriptive statistics (mean ± STD) of the studied sleep variables during the entire period of data collection including all soccer players, and during the night after a soccer night are shown in Table 1.

**Table 1:** Descriptive statistics for the subjective sleep patterns, based on the total period of 4,440 nights of data in 21 female football players, and the night after completing full soccer matches (252 nights), respectively.

Sleep variable	Mean (±STD)	Mean game night (±STD)
SSH- subjective sleep duration (h)	08:08 (±00:59)	07:59 (±01:07)
SLQ- subjective sleep quality (Number)	3.20 (±0.73)	3.17 (±0.73) – 63%

STD, standard deviation; SSH, subjective sleep duration; SLQ, subjective sleep quality.

The football players' subjective sleep measured by the PMSys online sport logging system showed that the mean SSH was 8 h and 7 min (±1:00) and their SLQ was 3.2 (±.74) in the control period (Exp1). In the experiment period the players' SSH was 8 h and 7 min (±01:01) and their SLQ was 3.18 (±.72). Table 2 shows the results from the MANOVA and ANOVA analyses investigating effects of type of days/nights (Type) and the control and experiment period (Exp).

**Table 2:** Multivariate and univariate ANOVA with false discovery rate adjusted *p*-values using FFMANOVA for validation of changes in subjective sleep variables on days close to football games (type) and the experimental periods (Exp) across all variables and within each variable.

MANOVA across all variables			ANOVA within each variable	
Variable	DF	*P*-value	SSH	SLQ
Type	3	**0,000**	**0,000**	**0,004**
Exp	1	**0,000**	**0,000**	**0,027**
Type*Exp	3	0,174	0,142	0,922
Residuals	1,461			

DF = degrees of freedom, Type = days that was not close to a game (TN), day before game (NBG), gameday (GN), gameday +1 (GN1) or gameday + 2 (GN2), SSH, Subjective sleep duration, hours and ½ hours; SLQ, Sleep quality, 1 = insomnia, 5 = excellent sleep.

The bold values represents statistical significant values.

For both subjective sleep variables (SSH and SLQ) there were significant main effects of type of night (Type) and significant main effects of experimental period (Exp). The interaction effect between type of night (Type) and experimental period (Exp) was not statistical significance.

**Figure 1:**
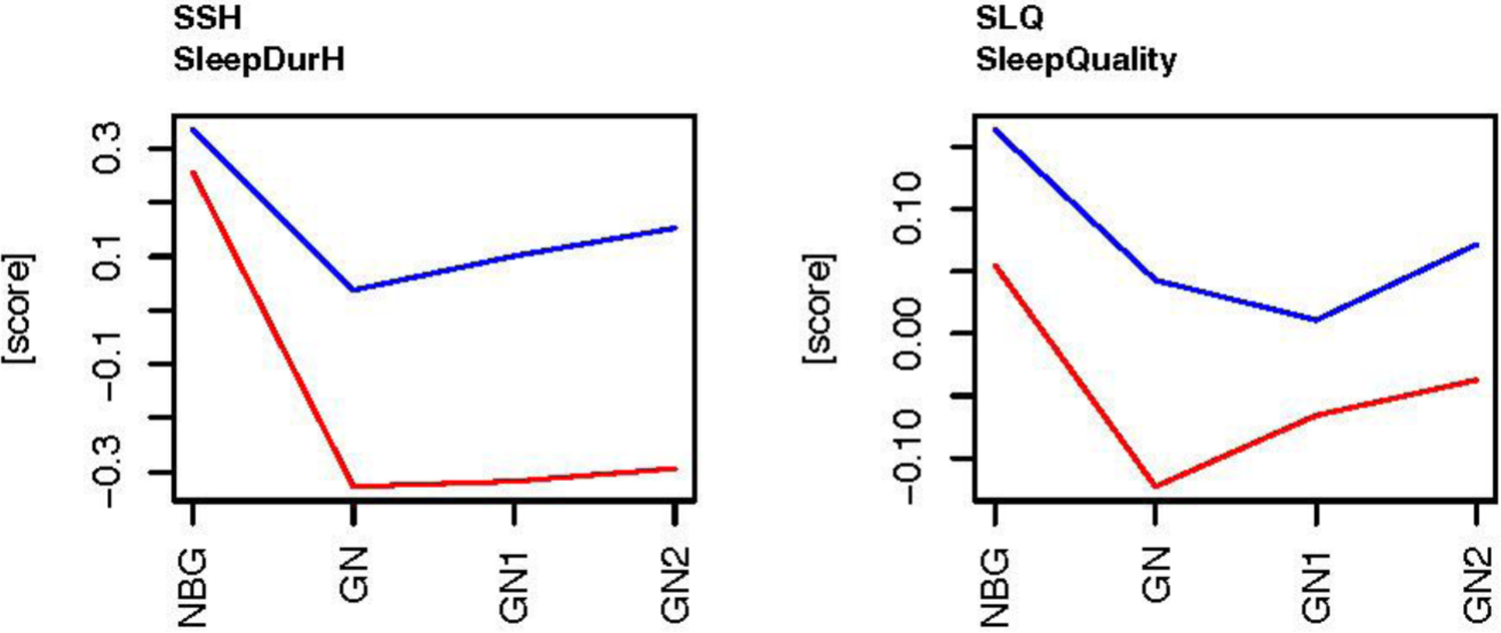
The subjective sleep data on days related to football games during the control period Exp1 (red) before BEMER, and the experiment period Exp3 (blue), the BEMER period. Subjective sleep duration (SSH, hours and ½ hours), Sleep quality (SLQ, 1 = insomnia, 5 = excellent sleep).

Figure 1 shows the subjective sleep variables conducted in the current study, subjective sleep duration (SSH) and subjective sleep quality (SLQ). The data display each type of nights as a difference to the players' means of the ordinary training days (baseline) that were not close to a game, as calculated for each player within each experimental period. The baseline (0 on the y-axis) is means in the control period (Exp1) and experiment period (Exp3), and the graphs show their values on night before gameday, gameday, gameday +1, and gameday +2, compared to their baseline in the control period (EXP1) and experiment period (Exp3).

There are main effects on type of night, where SSH and SLQ are significantly higher on the nights before a game (NBG) than on game nights (GN) (Table 3 and Figure 1), and there are main effects of the players' SSH and SLQ scores over the experiment periods (Exp1 and Exp3), where SSH is higher on game nights (GN) and the nights following game nights in the experiment period (Exp3) compared with the control period (Exp1). The SLQ is higher on all days related to football games in the experiment period (Exp3) compared with the control period (Exp1). The SSH and SLQ scores are also above the players' baselines on all nights related to football games in the experiment period (Exp3), whereas the variables are below the players' baselines in the control period (Exp1) on game nights (GN) and the nights following game nights (GN1 and GN2).

**Table 3:** The means, standard deviation, maximum and minimum values of the use of BEMER (BMT) and their subjective experience of possible experienced effects from the BEMER therapy on SRC, SSL, and SMC.

	BMT	SRC	SSL	SMC
Mean	595.7	4.48	4.24	4.29
STD	252.9	.98	1.41	.85
Maximum	983	6	7	6
Minimum	47	2	1	2

BMT, time spent on BEMER therapy; STD, standard deviation; SRC, Subjective restitution; SSL, subjective sleep; SMC, subjective muscle soreness.

The soccer players subjective experience of possible experienced effects from the BEMER therapy on their restitution (Subjective restitution-SRC), their sleep (Subjective sleep-SSL), and their experienced effect on their muscle soreness (Subjective muscle soreness-SMC), after completing the study are presented in Table 3.

Table 3 shows that the soccer players used BEMER therapy for 595.7 h on average during the experiment period, and that their subjective experienced effect of BEMER therapy on their restitution (SRC), their sleep (SSL), and their experienced effect on their muscle soreness (SMC), respectively were 4.48, 4.24, and 4.29.

## 4 Discussion

The aim of the current study was to investigate if Bio-Electro-Magnetic-Energy-Regulation (BEMER) therapy affects subjective sleep among elite players in Norwegian womeńs elite football. The hypothesis of the current study predicted that BEMER therapy would affect the players' subjective sleep on game nights and the nights following game days. The hypothesis was confirmed as the collected subjective data and the analyses show that subjective sleep duration and subjective sleep quality significantly improved in the experiment period compared to the control period.

### 4.1 Subjective sleep is improved by BEMER therapy

The results in the current study found that subjective measured sleep duration and sleep quality decreases significantly on game nights in both the control- and the experiment period (Table 2 and Figure 1). The significant decrease in sleep status on game nights paradoxically occurs in a situation where the elite women's football players need effective recovery caused by the extraordinary loads from the game. The decrease in the players' sleep status when their recovery needs are raised, represents a sleep disturbance (33, 34). A possible explanation is that the sleep disturbance is caused by the extraordinary physical and psychological loads on game days, causing emotional arousals and physical symptoms related to inflammation processes caused by muscle ruptures (21, 29, 33, 34, 38, 63). Interestingly, the players' sleep status on days following football games is significantly below their baseline sleep status for at least 3 days after game days in the control period (Table 2 and Figure 1). Interestingly, the recovery time for football players to achieve metabolic homeostasis after football games is found to be until 72–96 h (31, 64). Thus, the results in the current study show that the players' sleep status is significantly below their baseline sleep status on nights related to football games before the introduction of BEMER therapy. Therefore, it is reason to believe that the players in the current study are not fully recovered in the days after football games in the control period (Table 2 and Figure 1).

After the introduction of BEMER therapy the players subjective sleep duration and sleep quality were significantly increased compared to the control period on game nights and the following two nights (Table 2 and Figure 1). Both the players' subjective sleep duration and sleep quality were above the players' baseline sleep status on general training days on both game nights and the following two nights. The results in the current study also show that the players subjective sleep duration improves every night in the nights following game nights after the introduction of BEMER therapy.

Accordingly, after the introduction of BEMER therapy the players' subjective sleep quality does not drop below their baseline sleep status on game nights and increases significantly on the second night after game night (Figure 1). Before the introduction of BEMER therapy the players sleep quality increased the two days following games, but from a score significantly below their baseline sleep status on general training days (Figure 1). Thus, it might be because the players needed to catch up their sleep on days following game nights to become fully recovered.

A potential explanation of the current results might be that the players' recovery has improved in the experiment period compared to the control period. BEMER therapy treatment is found to improve vasomotion (45, 46, 49), and when blood perfusion improves it results in improved performance recovery (50). The recovery improves because of increased nutrition and oxygen delivery to cells, and transportation of waste and carbon dioxide from metabolism away from the cells (53). The possible effect on the players' vasomotion might be the reason why BEMER influences the players ability to gain more normal sleep after extraordinary loads such as football games (27).

In contrast to other studies that detected no change or a decrease in athletes' sleep status pre-competitions (65), the results in the current study show that the elite women's football players' sleep status on the nights before football games are significantly above their baseline sleep status (Table 2 and Figure 1). The current results are valid for both the control- and experiment period. The results indicate that the players are aware of their need to gain optimal sleep before football games and take actions to be fully recovered and ready for the game.

The playerś subjective experiences of possible experienced effects from the BEMER therapy treatment at the end of the intervention also show that the players are well above average satisfied with their experienced effects on their restitution (SRC), their sleep (SSL), and on their muscle soreness (SMC).

The results in the current study are in line with the results in a recent study that investigated effects from BEMER therapy on objective detected sleep, which found that total sleep time and most sleep stages significantly increased (27). Thus, the results in the current study show that BEMER therapy improves the elite women's football players subjective sleep duration and sleep quality on game nights and the nights following game nights.

### 4.2 Conclusion, strengths and limitations

Sleep is key in the recovery process as it helps regenerate important psychological and physiological functions. Since sleep is found to be disturbed after football matches and intensive football sessions, BEMER therapy treatment may be an effective tool to improve the recovery process. Especially during international tournaments football players need tools that can improve their recovery (66). The current results are especially interesting since elite athletes normally have a well-developed microcirculation caused by the amount of aerobic training they have completed (67). The strengths of the current study are the high numbers of daily observations over an extensive period, the unique subjects who are elite level players, and the confirmation of the findings detected by the objective measurements that were used to detect sleep in the same data collection (27). The current study also has limitations that should be kept in mind. The number of participants in the current study could have been larger and a control group would have strengthened the study. The relative low number of participants and data loss might have influenced the power of the statistical analyses.

## Data Availability

The raw data supporting the conclusions of this article will be made available by the authors, without undue reservation.

## References

[1] Bonnar D Bartel K Kakoschke N Lang C. Sleep interventions designed to improve athletic performance and recovery: a systematic review of current approaches. Sports Med. (2018) 48(3):683–703. 10.1007/s40279-017-0832-x 29352373

[2] Hrozanova M. Habitual sleep in junior athletes: associations with mental and physical stress loads (dissertation thesis). Norwegian University of Science and Technology, NTNU (2021).

[3] Venter RE. Role of sleep in performance and recovery of athletes: a review article. S Afr J Res Sport Phys Educ Recreation. (2012) 34(1):167–84.

[4] Manzar MD Zannat W Hussain ME. Sleep and physiological systems: a functional perspective. Biol Rhythm Res. (2015) 46:195–206. 10.1080/09291016.2014.966504

[5] Palmer CA Alfano CA. Sleep and emotion regulation: an organizing, integrative review. Sleep Med Rev. (2017) 31:6–16. 10.1016/j.smrv.2015.12.006 26899742

[6] Zee PC Badr MS Kushida C Mullington JM Pack AI Parthasarathy S Strategic opportunities in sleep and circadian research: report of the joint task force of the sleep research society and American academy of sleep medicine. Sleep. (2014) 37:219–27. 10.5665/sleep.3384 24501434 PMC3900611

[7] O’Donnell SL Beaven CM Jacobson GM Bird S Driller MW. Melatonin and sleep responses following exercise in elite female athletes. Sport Exerc Sci N.Z. (2019) 3:23–33. 10.36905/jses.2019.02.02

[8] O’Donnell S Beaven CM Driller MW. From pillow to podium: a review on understanding sleep for elite athletes. Nat Sci Sleep. (2018) 10:243–53. 10.2147/NSS.S158598 30197545 PMC6112797

[9] Roberts SSH Wei-Peng T Warmington SA. Effects of training and competition on the sleep of elite athletes: a systematic review and meta-analysis. Br J Sports Med. (2019) 53:513–22. 10.1136/bjsports-2018-099322 30217831

[10] Harvey AG Stinson K Whitaker KL Moskovitz D Virk H. The subjective meaning of sleep quality: a comparison of individuals with and without insomnia. Sleep. (2008) 31:383–93. 10.1093/sleep/31.3.383 18363315 PMC2276747

[11] Craven J McCartney D Desbrow B Sabapathy S Bellinger P Roberts L Effects of acute sleep loss on physical performance: a systematic and meta-analytical review. Sports Med. (2022) 52(11):2669–90. 10.1007/s40279-022-01706-y 35708888 PMC9584849

[12] Doherty R Madigan SM Nevill A Warrington G Ellis JG. The sleep and recovery practices of athletes. Nutrients. (2021) 13(4):1330. 10.3390/nu13041330 33920560 PMC8072992

[13] Hrozanova M Moen F Myhre K Klöckner C Pallesen S. Habitual sleep patterns of junior elite athletes in cross-country skiing and biathlon: a descriptive study. Cogent Med. (2018) 5(1):1–13. 10.1080/2331205X.2018.1548549

[14] Moen F Olsen M Hrozanova M. Associations between sleep patterns and performance development among Norwegian chess players. Front Psychol. (2020) 11:1855. 10.3389/fpsyg.2020.01855 32849090 PMC7401575

[15] Hrozanova M Klöckner CA Sandbakk Ø Pallesen S Moen F. Reciprocal associations between sleep, mental strain and training load in junior endurance athletes, and the role of poor subjective sleep quality. Front Psychol. (2020) 11:545581. 10.3389/fpsyg.2020.545581 33154725 PMC7586313

[16] Marqués-Jiménez D Calleja-González J Arratibel I Delextrat A Terrados N. Fatigue and recovery in soccer: evidence and challenges. Open Sports Sci J Bentham Open. (2017) 10(1):52–70. 10.2174/1875399X01710010052

[17] Moen F Olsen M Halmøy G Hrozanova M. Variations in elite female soccer Players’ sleep, and associations with perceived fatigue and soccer games. Front Sports Act Living. (2021) 3:694537. 10.3389/fspor.2021.694537 34514385 PMC8424084

[18] Baptista I Winther AK Johansen D Randers MB Pedersen S Pettersen SA. The variability of physical match demands in elite women’s football. Sci Med Footb. (2022) 12:1–7. 10.1080/24733938.2022.2027999 35060844

[19] Díaz-García J Filipas L La Torre A Gómez-Rivera J Rubio-Morales A García-Calvo T. Mental fatigue changes from regular season to play-offs in semiprofessional soccer: a comparison by training days. Scand J Med Sci Sports. (2023) 33(5):712–24. 10.1111/sms.14301 36601789

[20] Fédération Internationale de Football Association (FIFA). Physical analysis of the FIFA Women’s World Cup France 2019. (2019). Available online at: https://digitalhub.fifa.com/m/4f40a98140d305e2/original/zijqly4oednqa5gffgaz-pdf.pdf (Accessed January 10, 2022).

[21] Scott D Haigh J Lovell R. Physical characteristics and match performances in women’s international versus domestic-level football players: a 2-year, league-wide study. Sci Med Football. (2020) 4(3):211–5. 10.1080/24733938.2020.1745265

[22] UEFA. Champions League Technical Report 2021. (2021). Available online at: https://uefatechnicalreports.com/ucl-2021 (Accessed October 23, 2021).

[23] Smith MR Thompson C Marcora SM Skorski S Meyer T Coutts AJ. Mental fatigue and soccer: current knowledge and future directions. Sports Med. (2018) 48:1525–32. 10.1007/s40279-018-0908-2 29623604

[24] Williams AM. Perceptual skill in soccer: implications for talent identification and development. J Sports Sci. (2000) 18(9):737–50. 10.1080/02640410050120113 11043899

[25] Jordet G Aksum KM Pedersen DN Walvekar A Trivedi A McCall A Scanning, contextual factors, and association with performance in English premier league footballers: an investigation across a season. Front. Psychol. (2020) 11:553813. 10.3389/fpsyg.2020.553813 33123039 PMC7573254

[26] Datson N. An analysis of the physical demands of international female soccer match-play and the physical characteristics of elite players (Dissertation thesis). Liverpool John Moores University, Liverpool, UK (2016).

[27] Moen F Pettersen SA Gjertsås K Vatn M Ravenhorst M Kvålsvoll A The effect of bio-electro-magnetic-energyregulation therapy on sleep duration and sleep quality among elite players in Norwegian women’s football. Front Psychol. (2023) 14:1230281. 10.3389/fpsyg.2023.1230281 PMC10443099 37614490

[28] Nédélec M McCall A Carling C Legall F Berthoin S Dupont G. Recovery in soccer: part I—post-match fatigue and time course of recovery. Sports Med. (2012) 42(12):997–1015. 10.2165/11635270-000000000-00000 23046224

[29] Nédélec M McCall A Carling C Legall F Berthoin S Dupont G. Recovery in soccer: part ii-recovery strategies. Sports Med. (2013) 43(1):9–22. 10.1007/s40279-012-0002-0 23315753

[30] Peake JM Neubauer O Della Gatta PA Nosaka K. Muscle damage and inflammation during recovery from exercise. J Appl Physiol (1985). (2017) 122(3):559–70. 10.1152/japplphysiol.00971.2016 28035017

[31] Ispirlidis I Fatouros IG Jamurtas AZ Nikolaidis MG Michailidis I Douroudos I Time-course of changes in inflammatory and performance responses following a soccer game. Clin J Sport Med. (2008) 18:423–31. 10.1097/JSM.0b013e3181818e0b 18806550

[32] Nédélec M Halson S Abaidia AE Ahmaidi S Dupont G. Stress, sleep and recovery in elite soccer: a critical review of the literature. Sports Med. (2015) 45(10):1387–400. 10.1007/s40279-015-0358-z 26206724

[33] Fullagar HH Skorski S Duffield R Hammes D Coutts AJ Meyer T. Sleep and athletic performance: the effects of sleep loss on exercise performance, and physiological and cognitive responses to exercise. Sports Med. (2015) 45(2):161–86. 10.1007/s40279-014-0260-0 25315456

[34] Fullagar HHK Skorski S Duffield R Julian R Bartlett J Meyer T. Impaired sleep and recovery after night matches in elite football players. J Sports Sci. (2016) 34(14):1333–9. 10.1080/02640414.2015.1135249 26750446

[35] Silva AC Amaral AS Guerreiro R Silva A DeMello MT DaSilva SG Elite soccer athlete’s sleep: a literature review. Apunt Sport Med. (2022) 57:100373. 10.3390/app12062791

[36] Carriço S Skorski S Duffield R Mendes B Calvete F Meyer T. Post-match sleeping behavior based on match scheduling over a season in elite football players. Sci Med Footb. (2018) 2(1):9–15. 10.1080/24733938.2017.1403036

[37] Biggins M Purtill H Fowler P Bender A Sullivan KO Samuels C Sleep, health, and well-being in elite athletes from different sports, before, during, and after international competition. Phys Sportsmed. (2020) 30:1–9. 10.1080/00913847.2020.1850149 33251907

[38] de Hoyo M Cohen DD Sañudo B Carrasco L Álvarez-Mesa A Ojo JJ Influence of football match time–motion parameters on recovery time course of muscle damage and jump ability. J Sports Sci. (2016) 34(14):1363–70. 10.1080/02640414.2016.1150603 26930226

[39] Knufinke M Nieuwenhuys A Geurts SAE Møst EIS Maase K Moen MH Train hard, sleep well? Perceived training load, sleep quantity and sleep stage distribution in elite level athletes. J Sci Med Sport. (2018) 21(4):427–32. 10.1016/j.jsams.2017.07.003 28754605

[40] Cunha LA Costa JA Marques EA Brito J Lastella M Figueiredo P. The impact of sleep interventions on athletic performance: a systematic review. Sports Med—Open. (2023) 9(1):58. 10.1186/s40798-023-00599-z 37462808 PMC10354314

[41] Mah CD Mah KE Kezirian EJ Dement WC. The effects of sleep extension on the athletic performance of collegiate basketball players. Sleep. (2011) 34(7):943–50. 10.5665/SLEEP.1132 21731144 PMC3119836

[42] Charest J Grandner MA. Sleep and athletic performance: impacts on physical performance, mental performance, injury risk and recovery, and mental health. Sleep Med Clin. (2020) 15(1):41–57. 10.1016/j.jsmc.2019.11.005 32005349 PMC9960533

[43] Reilly T Ekblom B. The use of recovery methods post-exercise. J Sports Sci. (2005) 23:619–27. 10.1080/02640410400021302 16195010

[44] Basset CAL. Beneficial effects of electromagnetic fields. J Cell Biochem. (1993) 51:387–93. 10.1002/jcb.2400510402 8496242

[45] Bohn W. The technological development history and current significance of the “physical BEMER® vascular therapy” in medicine. J Complementary Integr Med. (2013) 10(Suppl):S1–3. 10.1515/jcim-2013-0036 24021601

[46] Bohn W Hess L Burger R. The effects of the “physical BEMER® vascular therapy,” a method for the physical stimulation of the vasomotion of precapillary micro vessels in case of impaired microcirculation, on sleep, pain and quality of life of patients with different clinical pictures on the basis of three scientifically validated scales. J Complementary Integr Med. (2013) 10(Suppl):5–12. 10.1515/jcim-2013-0037 23940071

[47] Ross CL Zhou Y McCall CE Soker S Criswell TL. The use of pulsed electromagnetic field to modulate inflammation and improve tissue regeneration: a review. Bioelectricity. (2019) 1(4):247–59. 10.1089/bioe.2019.0026 34471827 PMC8370292

[48] Intaglietta M. Vasomotion and flowmotion: physiological mechanisms and clinical evidence. Vasc Med Rev, vmr. (1990) 1(2):101–12. 10.1177/1358836X9000100202

[49] Klopp RC. Mikrozirkulation im Focus der Forschung. Mediquant Verlag (2017).

[50] Borne R Hausswirth C Bieuzen F. Relationship between blood flow and performance recovery: a randomized, placebo-controlled study. Int J Sports Physiol Perform. (2017) 12(2):152–60. 10.1123/ijspp.2015-0779 27139812

[51] Romero SA Minson CT Halliwill JR. The cardiovascular system after exercise. J Appl Physiol. (2017) 122(4):925–32. 10.1152/japplphysiol.00802.2016 28153943 PMC5407206

[52] Opondo MA Sarma S Levine BD. The cardiovascular physiology of sports and exercise. Clin Sports Med. (2015) 34:391–404. 10.1016/j.csm.2015.03.004 26100417

[53] Simons PC. Microcirculation of Blood- What Everyone Should Know About Physical Vascular Therapy: The Next Generation of Medicine? Norderstedt: Herstellung und Verlag: BoD-Books on demand (2020).

[54] Pal D Rudraiah N Devanathan RA. Couple stress model of blood flow in the microcirculation. Bltn Mathcal Biology. (1988) 50:329–44. 10.1007/BF02459703 3219442

[55] BEMER Group. Sports Recovery. (2021). Available online at: https://life.bemergroup.com/sports-recovery/ (Accessed April 07, 2021).

[56] BEMER Group. User Manual. (2020). Available online at: https://life.bemergroup.com/download/user-manual/ (Accessed April 08, 2021).

[57] Lockie R. Does pulsed electromagnetic field (PEMF) therapy have application to athletic performance? NSCA Coach. (2021) 7(4):12–6. Available online at: https://www.nsca.com/education/articles/nsca-coach/pulsed-electromagnetic-field-therapy-to-athletic-performance/

[58] Thambawita V Hicks SA Borgli H Stensland HK Jha D Svensen MK Pmdata: a sports logging dataset. Proceedings of the 11th ACM Multimedia Systems Conference (pp. 231-236) (2020).

[59] Moen B Oust A Langsrud Ø Dorrell N Marsden GL Hinds J Explorative multifactor approach for investigating global survival mechanisms of Campylobacter jejuni under environmental conditions. Appl Environ Microbiol. (2005) 71(4):2086–94. 10.1128/AEM.71.4.2086-2094.2005 15812042 PMC1082531

[60] Langsrud Ø. ANOVA For unbalanced data: use type II instead of type III sums of squares. Stat Comput. (2003) 13:163–7. 10.1023/A:1023260610025

[61] Langsrud Ø. Rotation tests. Stat Comput. (2005) 15:53–60. 10.1007/s11222-005-4789-5

[62] Langsrud Ø Jørgensen K Ofstad R Næs T. Analyzing designed experiments with multiple responses. J Appl Stat. (2007) 34(10):1275–96. 10.1080/02664760701594246

[63] Winther AK Baptista I Pedersen S Randers MB Johansen D Krustrup P Position specific physical performance and running intensity fluctuations in elite women’s football. Scand J Med Sci Sports. (2022) 32(Suppl 1):105–14. 10.1111/sms.14105 34825736

[64] Ascensão A Rebelo A Oliveira E Marques F Pereira L Magalhães J. Biochemical impact of a soccer match: analysis of oxidative stress and muscle damage markers throughout recovery. Clin Biochem. (2008) 41:841–51. 10.1016/j.clinbiochem.2008.04.008 18457670

[65] Gupta L Morgan K Gilchrist S. Does elite sport degrade sleep quality? A systematic review. Sports Med. (2017) 47:1317–33. 10.1007/s40279-016-0650-6 27900583 PMC5488138

[66] Lastella M Roach GD Sargent C. Travel fatigue and sleep/wake behaviors of professional soccer players during international competition. Sleep Health. (2019) 5(2):141–7. 10.1016/j.sleh.2018.10.013 30928113

[67] Liu Y Christensen PM Hellsten Y Gliemann L. Effects of exercise training intensity and duration on skeletal muscle capillarization in healthy subjects: a meta-analysis. Med Sci Sports Exercise. (2022) 54(10):1714–28. 10.1249/MSS.0000000000002955 35522254

